# Supplementary material for: Derivation of stationary distributions of biochemical reaction networks via structure transformation
Source: Commun Biol. 2021 May 24;4:620. doi: 10.1038/s42003-021-02117-x (PMC8144570; doi:10.1038/s42003-021-02117-x)
Supplement: Supplementary file 2 — Supplementary Information [file 42003_2021_2117_MOESM2_ESM.pdf]

Supplementary information: Derivation of stationary distributions of biochemical reaction networks via structure transformation

Hyukpyo Hong,[1,2,*] Jinsu Kim,[3,4,*] M Ali Al-Radhawi,[5] Eduardo D. Sontag,[5,6,7,8,9] and Jae Kyoung Kim[1,2,†]

[1]*Department of Mathematical Sciences, Korea Advanced Institute of Science and Technology, Daejeon 34141, Republic of Korea*

[2]*Biomedical Mathematics Group, Institute for Basic Science, Daejeon 34126, Republic of Korea*

[3]*Department of Mathematics, University of California, Irvine, CA 92697, United States*

[4]*NSF-Simons Center for Multiscale Cell Fate Research, University of California, Irvine, CA 92697, United States*

[5]*Department of Electrical and Computer Engineering, Northeastern University, Boston, MA 02115, United States*

[6]*Department of Bioengineering, Northeastern University, Boston, MA 02115, United States*

[7]*Department of Mathematics, Northeastern University, Boston, MA 02115, United States*

[8]*Department of Chemical Engineering, Northeastern University, Boston, MA 02115, United States*

[9]*Laboratory of Systems Pharmacology, Program in Therapeutics Science, Harvard Medical School, Boston, MA 02115, United States*

SUPPLEMENTARY NOTE 1: NETWORK TRANSLATION PRESERVES THE STOCHASTIC DYNAMICS

Let $\{\mathcal{S},\mathcal{C},\mathcal{R}\}$ and $\{\mathcal{S},\tilde{\mathcal{C}},\tilde{\mathcal{R}}\}$ be an original network and a translated network, respectively. Then, from the definition of network translation,

$$\sum_{k:\nu'_k-\nu_k=\gamma} \lambda_k(\mathbf{n}) = \sum_{\tilde{k}:\tilde{\nu}'_{\tilde{k}}-\tilde{\nu}_{\tilde{k}}=\gamma} \tilde{\lambda}_{\tilde{k}}(\mathbf{n}) \quad \text{for each vector } \gamma \in \mathbb{Z}^d, \tag{S1}$$

where λ_k and $\tilde{\lambda}_{\tilde{k}}$ are the propensities of stochastic models (i.e., continuous-time Markov chains) for $\{\mathcal{S},\mathcal{C},\mathcal{R}\}$ and $\{\mathcal{S},\tilde{\mathcal{C}},\tilde{\mathcal{R}}\}$, respectively. Let $p_{\mathbf{n}}(t)$ and $\tilde{p}_{\mathbf{n}}(t)$ be the probabilities of the continuous-time Markov chains associated with $\{\mathcal{S},\mathcal{C},\mathcal{R}\}$ and $\{\mathcal{S},\tilde{\mathcal{C}},\tilde{\mathcal{R}}\}$ being at state $\mathbf{n}$, respectively. Then, $p_{\mathbf{n}}(t)$ is the solution of CME given by:

$$\begin{aligned}
\frac{dp_{\mathbf{n}}}{dt} &= \sum_{k=1}^{r} \lambda_k(\mathbf{n}-(\nu'_k-\nu_k))p_{\mathbf{n}-(\nu'_k-\nu_k)} - \sum_{k=1}^{r} \lambda_k(\mathbf{n})p_{\mathbf{n}} \\
&= \sum_{\gamma\in\mathbb{Z}^d} \sum_{k:\nu'_k-\nu_k=\gamma} \lambda_k(\mathbf{n}-(\nu'_k-\nu_k))p_{\mathbf{n}-(\nu'_k-\nu_k)} - \sum_{\gamma\in\mathbb{Z}^d} \sum_{k:\nu'_k-\nu_k=\gamma} \lambda_k(\mathbf{n})p_{\mathbf{n}} \\
&= \sum_{\gamma\in\mathbb{Z}^d} \sum_{\tilde{k}:\tilde{\nu}'_{\tilde{k}}-\tilde{\nu}_{\tilde{k}}=\gamma} \tilde{\lambda}_{\tilde{k}}(\mathbf{n}-(\tilde{\nu}'_{\tilde{k}}-\tilde{\nu}_{\tilde{k}}))p_{\mathbf{n}-(\tilde{\nu}'_{\tilde{k}}-\tilde{\nu}_{\tilde{k}})} - \sum_{\gamma\in\mathbb{Z}^d} \sum_{\tilde{k}:\tilde{\nu}'_{\tilde{k}}-\tilde{\nu}_{\tilde{k}}=\gamma} \tilde{\lambda}_{\tilde{k}}(\mathbf{n})p_{\mathbf{n}} \\
&= \sum_{\tilde{k}=1}^{\tilde{r}} \tilde{\lambda}_{\tilde{k}}(\mathbf{n}-(\tilde{\nu}'_{\tilde{k}}-\tilde{\nu}_{\tilde{k}}))p_{\mathbf{n}-(\tilde{\nu}'_{\tilde{k}}-\tilde{\nu}_{\tilde{k}})} - \sum_{\tilde{k}=1}^{\tilde{r}} \tilde{\lambda}_{\tilde{k}}(\mathbf{n})p_{\mathbf{n}},
\end{aligned}$$

where r and $\tilde{r}$ are the total numbers of reactions in the original and the translated reaction networks, respectively, and the third equality follows from Eq. (S1). Thus, $p_{\mathbf{n}}(t)$ and $\tilde{p}_{\mathbf{n}}(t)$ are the solution of the same CME because the

* These authors contributed equally

† jaekkim@kaist.ac.kr

CME associated with the translated network is given by

$$\frac{d\tilde{p}_{\mathbf{n}}}{dt} = \sum_{\tilde{k}=1}^{\tilde{r}} \tilde{\lambda}_{\tilde{k}}(\mathbf{n} - (\tilde{\nu}'_{\tilde{k}} - \tilde{\nu}_{\tilde{k}}))\tilde{p}_{\mathbf{n}-(\tilde{\nu}'_{\tilde{k}}-\tilde{\nu}_{\tilde{k}})} - \sum_{\tilde{k}=1}^{\tilde{r}} \tilde{\lambda}_{\tilde{k}}(\mathbf{n})\tilde{p}_{\mathbf{n}}.$$

Therefore, $p_{\mathbf{n}}(t) \equiv \tilde{p}_{\mathbf{n}}(t)$ for all $\mathbf{n} \in \mathbb{Z}^d_{\geq 0}$ as long as they have the same initial condition.

SUPPLEMENTARY NOTE 2: PROPENSITY FACTORIZATION

As we discussed in the main text, to derive a stationary distribution, all the propensities $\lambda_k(\mathbf{n})$ should be factorized as

$$\lambda_k(\mathbf{n}) = \kappa_k \theta(\mathbf{n})\omega(\mathbf{n} - \nu_k)\mathbf{1}_{\{\mathbf{n}\geq\nu_k\}} \tag{S2}$$

for some constants $\kappa_k > 0$ and functions $\theta(\mathbf{n}) > 0$ and $\omega(\mathbf{n}) \geq 0$ on a set $\Gamma = \{\mathbf{n} \mid \mathbf{n} \geq \mathbf{b}\}$, where $\lambda_k(\mathbf{n}) > 0$ if and only if $\mathbf{n} \geq \nu_k + \mathbf{b}$ in Γ meaning that $\nu_k + \mathbf{b}$ represents the minimal copy numbers for the reaction to occur. This factorization form generalizes the previous result, Eq. (32) in [2], which requires the following factorization:

$$\lambda_k(\mathbf{n}) = \kappa_k \frac{\zeta(\mathbf{n})}{\zeta(\mathbf{n} - \nu_k)}\mathbf{1}_{\{\mathbf{n}\geq\nu_k\}} \tag{S3}$$

for $\zeta : \mathbb{Z}^d_{\geq 0} \to \mathbb{R}_{>0}$ on $\mathbb{Z}^d_{\geq 0}$. Note that if $\omega(\mathbf{n}) = \frac{1}{\theta(\mathbf{n})}$ and $\Gamma = \mathbb{Z}^d_{\geq 0}$ (i.e., $\mathbf{b} = \mathbf{0}$), Eq. (S2) is equivalent to Eq. (S3).

Propensity factorization by solving recurrence relations

To obtain the desired factorization for given propensity functions, we need to solve recurrence relations. Here, we illustrate how to solve the following factorization equations of Fig. 1b.

$$\lambda_1(n_A, n_B) = \alpha_1 + \alpha_4 n_A = \kappa_1 \theta(n_A, n_B)\omega(n_A, n_B), \tag{S4}$$

$$\lambda_2(n_A, n_B) = \alpha_2 n_A = \kappa_2 \theta(n_A, n_B)\omega(n_A - 1, n_B)\mathbf{1}_{\{n_A\geq 1\}}, \text{ and} \tag{S5}$$

$$\lambda_3(n_A, n_B) = \alpha_3 n_A n_B = \kappa_3 \theta(n_A, n_B)\omega(n_A - 1, n_B - 1)\mathbf{1}_{\{n_A\geq 1, n_B\geq 1\}}. \tag{S6}$$

After setting $\kappa_k = \alpha_k$, we divide Eq. (S4) and Eq. (S5) by Eq. (S5) and Eq. (S6), respectively. Then we have $\omega(n_A, n_B) = (\frac{\alpha_4 n_A + \alpha_1}{\alpha_1 n_A})\omega(n_A - 1, n_B)$ and $\omega(n_A - 1, n_B) = \frac{1}{n_B}\omega(n_A - 1, n_B - 1)$. Solving these recurrence relations, we get $\omega(n_A, n_B) = \prod_{j=1}^{n_A}\left(\frac{\alpha_4 j + \alpha_1}{\alpha_1 j}\right)\frac{1}{n_B!}\omega(0,0)$. Then $\theta(n_A, n_B)$ is determined as $\frac{(\alpha_4 n_A + \alpha_1)}{\alpha_1 \omega(0,0)}\prod_{j=1}^{n_A}\left(\frac{\alpha_1 j}{\alpha_4 j + \alpha_1}\right)n_B!$ from the second equality in Eq. (S4). Note that this factorization cannot be accomplished by the previously identified form (Eq. (S3), $\lambda_k = \kappa_k \theta(\mathbf{n})\theta(\mathbf{n} - \nu_k)^{-1}\mathbf{1}_{\{\mathbf{n}\geq\nu_k\}}$), which requires $\omega(n_A, n_B) = \theta(n_A, n_B)^{-1}$, because Eq. (S4) implies $\omega(n_A, n_B) \neq \theta(n_A, n_B)^{-1}$. Here, $\omega(0,0)$ can be determined by requiring the sum of the stationary distribution $\sum_{\mathbf{n}} \pi(\mathbf{n})$ to be 1.

Propensities following generalized mass action kinetics can be factorized without solving recurrence relations

If the propensities follow a sort of generalized stochastic mass action kinetics, then their factorization can be easily obtained. In what follows, we use the convention that $\prod_{j=0}^{-1} a_j = 1$ for any $\{a_j\}$.

Theorem 1. *Let a biochemical reaction network* $\{\mathcal{S}, \mathcal{C}, \mathcal{R}\}$ *be given, and let* $\lambda_k(\mathbf{n})$ *be the propensity functions of the associated continuous-time Markov chain. Suppose that there exists a vector* $\mathbf{b}$ *such that for* $\mathbf{n} \in \Gamma = \{\mathbf{n} \mid \mathbf{n} \geq \mathbf{b}\}$*, we*

have $\lambda_k(\mathbf{n}) > 0$ if $\mathbf{n} \geq \nu_k + \mathbf{b}$ and $\lambda_k(\mathbf{n}) = 0$ otherwise for each $k = 1, 2, \ldots, r$. We further assume that there exist constants $\alpha_k > 0$ and functions $f_i : \mathbb{Z}_{\geq 0} \to \mathbb{R}_{\geq 0}, i = 1, \ldots, d$ such that $\lambda_k(\mathbf{n}) = \alpha_k \prod_{i=1}^{d} \prod_{j=0}^{\nu_{ki}-1} f_i(n_i - j)$ for $k = 1, \ldots, r$ and all $\mathbf{n} \in \Gamma$, where ν_{ki} is the ith entry of the source complex vector ν_k. Then $\lambda_k(\mathbf{n}) = \kappa_k \theta(\mathbf{n}) \omega(\mathbf{n} - \nu_k) \mathbf{1}_{\{\mathbf{n} \geq \nu_k\}}$ on the Γ with

$$\kappa_k := \alpha_k, \quad \theta(\mathbf{n}) := \prod_{i=1}^{d} \prod_{j=b_i+1}^{n_i} f_i(j), \quad \text{and} \quad \omega(\mathbf{n}) := \frac{1}{\theta(\mathbf{n})} \mathbf{1}_{\{\mathbf{n} \geq \mathbf{b}\}}. \tag{S7}$$

Proof. For $\mathbf{n} \in \Gamma$,

$$\begin{aligned}
\kappa_k \theta(\mathbf{n}) \omega(\mathbf{n} - \nu_k) \mathbf{1}_{\{\mathbf{n} \geq \nu_k\}} &= \alpha_k \frac{\prod_{i=1}^{d} \prod_{j=b_i+1}^{n_i} f_i(j)}{\prod_{i=1}^{d} \prod_{j=b_i+1}^{n_i - \nu_{ki}} f_i(j)} \mathbf{1}_{\{\mathbf{n} - \nu_k \geq \mathbf{b}\}} \mathbf{1}_{\{\mathbf{n} \geq \nu_k\}} \\
&= \alpha_k \prod_{i=1}^{d} \prod_{j=n_i - \nu_{ki}+1}^{n_i} f_i(j) \mathbf{1}_{\{n_i - \nu_{ki} \geq b_i\}} \\
&= \alpha_k \prod_{i=1}^{d} \prod_{j=0}^{\nu_{ki}-1} f_i(n_i - j) \mathbf{1}_{\{n_i - \nu_{ki} \geq b_i\}} \\
&= \begin{cases} \alpha_k \prod_{i=1}^{d} \prod_{j=0}^{\nu_{ki}-1} f_i(n_i - j) & \text{if } \mathbf{n} \geq \nu_k + \mathbf{b} \\ 0 & \text{otherwise} \end{cases} \\
&= \lambda_k(\mathbf{n}).
\end{aligned}$$

□

Remark 1. A similar condition was introduced by Eq. (27) in the previous study [2], which is a special case of the generalized mass action kinetics with $\mathbf{b} = 0$. Furthermore, the associated function $f_i(n_i)$ can be thought as the "rate of association" of the ith species as pointed out in [9]. Such rate of association of the species have been also used in order to generalize the Horn-Jackson-Feinberg theory to deterministic non-mass action system [11].

Remark 2. The example in Fig. 3g does not follow the generalized mass action kinetics because the propensity of the reaction $A \to A_P$, $\alpha_1 n_A + \alpha_2 n_A n_{A_P}$, depends on both n_A and n_{A_P}, but the latter is not supported in the source complex. Nevertheless, it can be re-expressed as $\alpha_1 n_A + \alpha_2 n_A (T_0 - n_A)$ using the conservation law: $n_A + n_{A_P} = T_0$ so it now follows the generalized mass action kinetics.

SUPPLEMENTARY NOTE 3: DERIVATION OF STATIONARY DISTRIBUTIONS

If a network is weakly reversible and has zero deficiency, the deterministic mass action model with any rate constants always possesses a CBE $\mathbf{c} \in \mathbb{R}^d_{>0}$ [4, 7, 8]. Then the existence of the propensity factorization ensures that the stationary distribution of the associated continuous-time Markov chain can be derived analytically. In what follows, $\mathbf{x} \not\geq \mathbf{y}$ means there is at least one i such that $x_i < y_i$.

Theorem 2. *For a given biochemical reaction network $\{\mathcal{S}, \mathcal{C}, \mathcal{R}\}$, suppose that there exists a complex balanced equilibrium $\mathbf{c} \in \mathbb{R}^d_{>0}$ for the deterministic mass action model for $\{\mathcal{S}, \mathcal{C}, \mathcal{R}\}$ with rate constants $\{\kappa_k\}$. Let $\lambda_k(\mathbf{n})$ be the propensity functions of the continuous-time Markov chain associated with the network $\{\mathcal{S}, \mathcal{C}, \mathcal{R}\}$. Suppose that there exists a vector $\mathbf{b}$ such that for $\mathbf{n} \in \Gamma = \{\mathbf{n} \mid \mathbf{n} \geq \mathbf{b}\}$, we have $\lambda_k(\mathbf{n}) > 0$ if $\mathbf{n} \geq \nu_k + \mathbf{b}$ and $\lambda_k(\mathbf{n}) = 0$ otherwise for each $k = 1, 2, \ldots, r$. Assume further that the propensity functions $\lambda_k(\mathbf{n})$ can be factorized as*

$$\lambda_k(\mathbf{n}) = \kappa_k \theta(\mathbf{n}) \omega(\mathbf{n} - \nu_k) \mathbf{1}_{\{\mathbf{n} \geq \nu_k\}} \text{ on } \Gamma = \{\mathbf{n} \mid \mathbf{n} \geq \mathbf{b}\}, \tag{S8}$$

where θ and ω are non-negative functions such that $\theta(\mathbf{n}) > 0$ if $\mathbf{n} \in \Gamma$. Then the Γ is a closed subset, and the stochastic model admits a stationary measure that can be written as

$$\pi(\mathbf{n}) = \begin{cases} \dfrac{\mathbf{c}^{\mathbf{n}}}{\theta(\mathbf{n})} & \textit{if } \mathbf{n} \in \Gamma, \\ 0 & \textit{if } \mathbf{n} \in \Gamma^c. \end{cases}$$

If π is summable over the state space, then the measure π can be normalized to be a stationary distribution.

Proof. We first show that Γ is closed, i.e., there is no transition from Γ to Γ^c. Assume that there exists a reaction from $\mathbf{n} \in \Gamma$ to $\mathbf{n} + \nu_k' - \nu_k \in \Gamma^c$ with $\lambda_k(\mathbf{n}) > 0$. This implies that $n_i + \nu_{ki}' - \nu_{ki} < b_i$ for some i, and thus $n_i < b_i - \nu_{ki}' + \nu_{ki} \leq b_i + \nu_{ki}$. That is, $\mathbf{n} \not\geq \nu_k + \mathbf{b}$, and $\mathbf{n} \geq \mathbf{b}$ since $\mathbf{n} \in \Gamma$. Therefore, $\lambda_k(\mathbf{n}) = 0$ from the definition of $\mathbf{b}$. This contradicts to $\lambda_k(\mathbf{n}) > 0$, so Γ is closed.

We turn to showing that the given π solves the CME at steady state:

$$\sum_k \lambda_k(\mathbf{n} - (\nu_k' - \nu_k))\pi(\mathbf{n} - (\nu_k' - \nu_k)) = \sum_k \lambda_k(\mathbf{n})\pi(\mathbf{n}) \quad \text{for all } \mathbf{n} \in \mathbb{Z}^d_{\geq 0}. \tag{S9}$$

Case 1: $\mathbf{n} \in \Gamma^c$. The right-hand side in Eq. (S9) is 0 as $\pi(\mathbf{n}) = 0$ for $\mathbf{n} \in \Gamma^c$. For the left-hand side in Eq. (S9), if $\mathbf{n} - (\nu_k' - \nu_k) \in \Gamma$, $\lambda_k(\mathbf{n} - (\nu_k' - \nu_k)) = 0$ because Γ is a closed set, and if $\mathbf{n} - (\nu_k' - \nu_k) \in \Gamma^c$, $\pi(\mathbf{n} - (\nu_k' - \nu_k)) = 0$ by its definition. Therefore, the left-hand side in Eq. (S9) is 0.
Case 2: $\mathbf{n} \in \Gamma$. For each $\mathbf{n} \in \Gamma$, we define $K_\Gamma(\mathbf{n}) = \{k \mid \mathbf{n} - (\nu_k' - \nu_k) \in \Gamma\}$. Then Eq. (S9) can be reduced into

$$\sum_{k \in K_\Gamma(\mathbf{n})} \lambda_k(\mathbf{n} - (\nu_k' - \nu_k))\pi(\mathbf{n} - (\nu_k' - \nu_k)) = \sum_k \lambda_k(\mathbf{n})\pi(\mathbf{n}) \quad \text{for all } \mathbf{n} \in \mathbb{Z}^d_{\geq 0} \tag{S10}$$

because $\pi(n - (\nu_k' - \nu_k)) = 0$ for each $k \in K_\Gamma(\mathbf{n})^c$. After the substitution of $\pi(\mathbf{n}) = \dfrac{\mathbf{c}^n}{\theta(\mathbf{n})}$ and Eq. (S8), the Eq. (S10) becomes

$$\begin{aligned} LHS &= \sum_{k \in K_\Gamma(\mathbf{n})} \kappa_k \frac{\mathbf{c}^{\mathbf{n}-(\nu_k'-\nu_k)}}{\theta(\mathbf{n} - (\nu_k' - \nu_k))} \theta(\mathbf{n} - (\nu_k' - \nu_k))\omega(\mathbf{n} - \nu_k')\mathbf{1}_{\{\mathbf{n} \geq \nu_k'\}} \\ &= \mathbf{c}^{\mathbf{n}} \sum_{k \in K_\Gamma(\mathbf{n})} \kappa_k \mathbf{c}^{\nu_k - \nu_k'}\omega(\mathbf{n} - \nu_k')\mathbf{1}_{\{\mathbf{n} \geq \nu_k'\}}, \end{aligned} \tag{S11}$$

$$RHS = \frac{\mathbf{c}^{\mathbf{n}}}{\theta(\mathbf{n})} \sum_k \kappa_k \theta(\mathbf{n})\omega(\mathbf{n} - \nu_k)\mathbf{1}_{\{\mathbf{n} \geq \nu_k\}} = \mathbf{c}^{\mathbf{n}} \sum_k \kappa_k \omega(\mathbf{n} - \nu_k)\mathbf{1}_{\{\mathbf{n} \geq \nu_k\}}. \tag{S12}$$

We can show that the RHS and LHS are equal by using that $\mathbf{c} \in \mathbb{R}^d_{>0}$ is a CBE and thus for any fixed complex $z \in \mathcal{C}$,

$$\sum_{k:\nu_k'=z} \kappa_k \mathbf{c}^{\nu_k} = \sum_{k:\nu_k=z} \kappa_k \mathbf{c}^{\nu_k}.$$

Multiplying $\mathbf{c}^{-z}\omega(\mathbf{n} - z)\mathbf{1}_{\{\mathbf{n} \geq z\}}$ on the both sides, we have

$$\begin{aligned} \sum_{k:\nu_k'=z} \kappa_k \mathbf{c}^{\nu_k - z}\omega(\mathbf{n} - z)\mathbf{1}_{\{\mathbf{n} \geq z\}} &= \sum_{k:\nu_k=z} \kappa_k \mathbf{c}^{\nu_k - z}\omega(\mathbf{n} - z)\mathbf{1}_{\{\mathbf{n} \geq z\}}, \\ \sum_{k:\nu_k'=z} \kappa_k \mathbf{c}^{\nu_k - \nu_k'}\omega(\mathbf{n} - \nu_k')\mathbf{1}_{\{\mathbf{n} \geq \nu_k'\}} &= \sum_{k:\nu_k=z} \kappa_k \omega(\mathbf{n} - \nu_k)\mathbf{1}_{\{\mathbf{n} \geq \nu_k\}}. \end{aligned}$$

Hence, by summing these up for all $z \in \mathcal{C}$ and multiplying $\mathbf{c}^{\mathbf{n}}$ both sides, we get

$$\mathbf{c}^{\mathbf{n}} \sum_k \kappa_k \mathbf{c}^{\nu_k - \nu_k'}\omega(\mathbf{n} - \nu_k')\mathbf{1}_{\{\mathbf{n} \geq \nu_k'\}} = \mathbf{c}^{\mathbf{n}} \sum_k \kappa_k \omega(\mathbf{n} - \nu_k)\mathbf{1}_{\{\mathbf{n} \geq \nu_k\}} \tag{S13}$$

because every reaction has exactly one complex as its source complex and product complex. Since the right hand side of Eq. (S13) is equal to the RHS (Eq. (S12)), we need to show that the left hand side of Eq. (S13) is equal to the LHS (Eq. (S11)).

If $k \in K_\Gamma(\mathbf{n})^c$, then $\mathbf{n} - (\nu'_k - \nu_k) \in \Gamma^c$ meaning that $n_i - \nu'_{ki} \le n_i - (\nu'_{ki} - \nu_{ki}) < b_i$ for some i and thus $\mathbf{n} - \nu'_k \in \Gamma^c$. Since the network has the complex balanced equilibrium, the network is weakly reversible [7]. Thus, there exists a reaction $\tilde{k}$ whose source complex $\nu_{\tilde{k}}$ is equal to ν'_k. For such $\tilde{k}$, by the definition of $\mathbf{b}$, $\lambda_{\tilde{k}}(\mathbf{n}) = \kappa_{\tilde{k}}\theta(\mathbf{n})\omega(\mathbf{n} - \nu_{\tilde{k}})\mathbf{1}_{\{\mathbf{n}\ge\nu_{\tilde{k}}\}} = 0$ because $\mathbf{n} \ge \mathbf{b}$ and $\mathbf{n} - \nu_{\tilde{k}} \in \Gamma^c$ (i.e., $\mathbf{n} - \nu_{\tilde{k}} \not\ge \mathbf{b}$). Since $\theta(\mathbf{n}) > 0$, furthermore, $\omega(\mathbf{n} - \nu_{\tilde{k}})\mathbf{1}_{\{\mathbf{n}\ge\nu_{\tilde{k}}\}} = \omega(\mathbf{n} - \nu'_k)\mathbf{1}_{\{\mathbf{n}\ge\nu'_k\}} = 0$. Hence, the left hand side of Eq. (S13) is equal to the Eq. (S11) and therefore, Eq. (S11) and Eq. (S12) are equal. □

Example 1. Here, we illustrate Theorem 2 with the translated EGFR network in Fig. 3a:

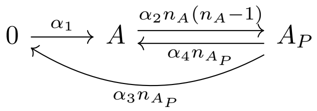

The CBE $\mathbf{c} = (c_A, c_{A_P})$ of the deterministic mass action model satisfies the balancing equations for the complex 0: $\alpha_3 c_{A_P} = \alpha_1$, for the complex A: $\alpha_1 + \alpha_4 c_{A_P} = \alpha_2 c_A$, and for the complex A_P : $\alpha_2 c_A = \alpha_3 c_{A_P} + \alpha_4 c_{A_P}$. Thus, $\mathbf{c} = (c_A, c_{A_P}) = (\frac{\alpha_1}{\alpha_2} + \frac{\alpha_1\alpha_4}{\alpha_2\alpha_3}, \frac{\alpha_1}{\alpha_3})$. For this example, $\mathbf{b} = (1, 0)$ since $\lambda_k(\mathbf{n}) > 0$ for $\mathbf{n} \ge \nu_k + (1, 0)$, and $\lambda_k(\mathbf{n}) = 0$ for $\mathbf{n}$ such that $\mathbf{n} \ge (1, 0)$ and $\mathbf{n} \not\ge \nu_k + (1, 0)$ for all $k = 1, \dots, 4$. Letting $f_A(n_A) = n_A(n_A - 1)$ and $f_{A_p} = n_{A_P}$, the condition in Theorem 1 holds. Therefore, the propensity factorization $\lambda_k(\mathbf{n}) = \kappa_k\theta(\mathbf{n})\omega(\mathbf{n} - \nu_k)\mathbf{1}_{\{\mathbf{n}\ge\nu_k\}}$ is given by

$$\kappa_k = \alpha_k, \quad \theta(n_A, n_{A_P}) = n_A!(n_A - 1)!n_{A_P}!$$

and

$$\omega(n_A, n_B) = \frac{1}{\theta(n_A, n_{A_P})}\mathbf{1}_{\{n_A\ge1\}} = \frac{1}{n_A!(n_A - 1)!n_{A_P}!}\mathbf{1}_{\{n_A\ge1\}}$$

for $(n_A, n_{A_P}) \in \Gamma = \{(n_A, n_{A_P}) \mid n_A \ge 1, n_{A_P} \ge 0\}$. By Theorem 2, a stationary measure $\pi(n_A, n_{A_P})$ is given by

$$\pi(n_A, n_{A_P}) = \begin{cases} M\dfrac{\left(\frac{\alpha_1}{\alpha_2} + \frac{\alpha_1\alpha_4}{\alpha_2\alpha_3}\right)^{n_A}\left(\frac{\alpha_1}{\alpha_3}\right)^{n_{A_P}}}{n_A!(n_A - 1)!n_{A_P}!} & \text{if } (n_A, n_{A_P}) \in \Gamma, \\ 0 & \text{if } (n_A, n_{A_P}) \in \Gamma^c. \end{cases}$$

where M is a normalizing constant. $\pi(n_A, n_{A_P})$ is indeed a stationary distribution because it is summable over Γ as follows:

$$\begin{aligned} \sum_{(n_A,n_{A_P})\in\Gamma} \frac{\left(\frac{\alpha_1}{\alpha_2} + \frac{\alpha_1\alpha_4}{\alpha_2\alpha_3}\right)^{n_A}\left(\frac{\alpha_1}{\alpha_3}\right)^{n_{A_P}}}{n_A!(n_A - 1)!n_{A_P}!} &\le \sum_{(n_A,n_{A_P})\in\Gamma} \frac{\left(\frac{\alpha_1}{\alpha_2} + \frac{\alpha_1\alpha_4}{\alpha_2\alpha_3}\right)^{n_A}\left(\frac{\alpha_1}{\alpha_3}\right)^{n_{A_P}}}{n_A!n_{A_P}!} \\ &\le \sum_{(n_A,n_{A_P})\in\mathbb{Z}^2_{\ge0}} \frac{\left(\frac{\alpha_1}{\alpha_2} + \frac{\alpha_1\alpha_4}{\alpha_2\alpha_3}\right)^{n_A}\left(\frac{\alpha_1}{\alpha_3}\right)^{n_{A_P}}}{n_A!n_{A_P}!} \\ &= \exp\left(\frac{\alpha_1}{\alpha_2} + \frac{\alpha_1\alpha_4}{\alpha_2\alpha_3} + \frac{\alpha_1}{\alpha_3}\right) < \infty. \end{aligned}$$

Moreover, since Γ is closed, the states $(n_A, n_{A_P}) \in \Gamma^c$ are transient as each state in Γ^c is reachable to Γ with either $0 \to A$ or $A_p \to A$. This implies that the stochastic process can visit the state $\mathbf{n} \in \Gamma^c$ only finitely many times. Indeed, if $(n_A, n_{A_P}) \in \Gamma^c$, i.e., $n_A = 0$, then only the reactions $0 \to A$, $A_P \to A$ and $A_P \to 0$ can occur in Γ^c. Thus, the process moves along one of the stoichiometric vectors $(1, 0), (1, -1)$, and $(0, -1)$, then it eventually escapes from

Γ^c to Γ and never comes back to Γ^c. Due to the transient states, Theorem 6.6, which is the most general version of the previous work [2], is not applicable to this example.

Let us compute the marginal means and variances of n_A and n_{A_P}. For n_{A_P}, the marginal distribution

$$\pi(n_{A_P}) = M_1 \frac{(\alpha_1/\alpha_3)^{n_{A_P}}}{n_{A_P}!}, \quad n_{A_P} \geq 0,$$

which is a Poisson distribution with rate $\frac{\alpha_1}{\alpha_3}$, so both the mean and the variance are $\frac{\alpha_1}{\alpha_3}$. Thus, the coefficient of variation (CV) is $\sqrt{\frac{\alpha_3}{\alpha_1}}$, and the Fano factor, defined as the variance divided by the mean, is always 1. For n_A, the marginal distribution

$$\pi(n_A) = M_2 \frac{u_0^{n_A}}{(n_A - 1)! n_A!}, \quad n_A \geq 1$$

where $u_0 = (\alpha_1/\alpha_2 + \alpha_1\alpha_4/\alpha_2\alpha_3)$, and M_2 is the normalizing constant.

$$M_2 = \left(\sum_{n_A=1}^{\infty} \frac{u_0^{n_A}}{n_A!(n_A - 1)!} \right)^{-1} = [\sqrt{u_0} I_1(2\sqrt{u_0})]^{-1}$$

where $I_m(x)$ is the modified Bessel function of the first kind. Then the mean and variance are given by

$$\mathbb{E}[n_A] = \sum_{n_A=1}^{\infty} n_A \pi(n_A) = \sqrt{u_0} \frac{I_0(2\sqrt{u_0})}{I_1(2\sqrt{u_0})},$$

$$\mathrm{Var}(n_A) = \mathbb{E}[n_A^2] - (\mathbb{E}[n_A])^2 = \sqrt{u_0} \frac{I_0(2\sqrt{u_0})}{I_1(2\sqrt{u_0})} + u_0 - u_0 \frac{I_0(2\sqrt{u_0})^2}{I_1(2\sqrt{u_0})^2}.$$

The CV and the Fano factor are also given by

$$\mathrm{CV} = \frac{\sqrt{\mathrm{Var}(n_A)}}{\mathbb{E}[n_A]} = \sqrt{\frac{\mathbb{E}[n_A^2]}{\mathbb{E}[n_A]^2} - 1} = \sqrt{\frac{1}{\sqrt{n_0}} \frac{I_1(2\sqrt{u_0})}{I_0(2\sqrt{u_0})} + \frac{I_0(2\sqrt{u_0})^2}{I_1(2\sqrt{u_0})^2} - 1},$$

$$\text{Fano factor} = \frac{\mathrm{Var}(n_A)}{\mathbb{E}[n_A]} = 1 + \sqrt{u_0} \frac{I_1(2\sqrt{u_0})}{I_0(2\sqrt{u_0})} - \sqrt{u_0} \frac{I_0(2\sqrt{u_0})}{I_1(2\sqrt{u_0})}.$$

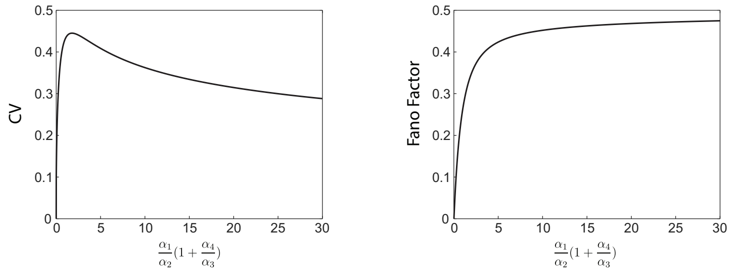

Supplementary Figure 1. The coefficient of variation (CV) and Fano factor of the stationary distribution of n_A in Fig. 3A can be determined by the single parameter $u_0 = \frac{\alpha_1}{\alpha_2}(1 + \frac{\alpha_4}{\alpha_3})$. The CV attains the maximum at $u_0 \approx 1.8$, and the Fano factor monotonically increases. Since the Fano factor is always less than 1, this distribution is sub-Poissonian.

Remark 3. By the basic Markov properties [10], Theorem 2 not only provides a stationary measure but also characterizes the status of states. Since $\pi(\mathbf{n}) > 0$ for each $\mathbf{n} \in \Gamma$, every state in Γ is recurrent as long as the process is non-explosive (i.e., well-defined for all time t). Since Γ is a closed set, furthermore, if a state $\mathbf{n} \in \Gamma^c$ is reachable to Γ, then $\mathbf{n}$ is transient. Another special case is that a state $\mathbf{n}$ is isolated state, i.e., $\lambda_k(\mathbf{n}) = 0$ for all k. In this case, the set of the single element $\{\mathbf{n}\}$ is an irreducible subset, and $\pi(\mathbf{n}) = 1$ on $\{\mathbf{n}\}$ is a stationary distribution, obviously.

Remark 4. This theorem does not require nor imply the irreducibility of Γ. If Γ is not irreducible then for an irreducible subset $\Gamma_g \subset \Gamma$, we consider a measure $\pi_{\Gamma_g} = \pi|_{\mathbf{n}\in\Gamma_g}$ restricted on Γ_g. This measure is still a stationary measure and can be a stationary distribution on Γ_g as long as π_{Γ_g} is summable over Γ_g. For instance, in the example in Fig. 3d, Γ is not an irreducible set, but a union of irreducible subsets $\Gamma_{T_0} = \{(x_1, x_2, x_3) \in \mathbb{Z}^3_{\geq 0} \mid x_1 \geq 1 \text{ and } x_1 + x_2 + x_3 = T_0\}$. Since the stationary measure π obtained by Theorem 2 is summable on Γ_{T_0} for each T_0, the restricted stationary measure $\pi_{\Gamma_{T_0}}$ can be normalized to be a stationary distribution on the irreducible subset. In this remark, we prove the irreducibility of $\{(x_1, x_2, x_3) \in \mathbb{Z}^3_{\geq 0} \mid x_1 \geq 1 \text{ and } x_1 + x_2 + x_3 = T_0\}$ for Fig. 3d. We say that for a Markov chain X, state y is *accessible* from state x and write $x \to_{\mathrm{a}} y$ if X can reach y starting from x with positive probability, that is, the probabilities $P(X(t) = y \mid X(0) = x) > 0$ for some $t > 0$. We say that states x and y *communicate* and write $x \leftrightarrow_{\mathrm{c}} y$ if $x \to_{\mathrm{a}} y$ and $y \to_{\mathrm{a}} x$. For the Markov chain $X(t)$ associated with a translated network, we denote by $\lambda_{C_1\to C_2}$ and η the propensity of a reaction $C_1 \to C_2$ and the stoichiometric vector of the reaction, respectively. Note that if $\lambda_{C_1\to C_2}(x) > 0$ then $x \to_{\mathrm{a}} x + \eta$ because for a sufficiently small Δt,

$$P(X(\Delta t) = x + \eta \mid X(0) = x) = \lambda_{C_1\to C_2}(x)\Delta t + o(\Delta t).$$

By using this, for the translated network in Fig. 3d, we have $(n_A, n_{A_P}, n_{A_{PP}}) \leftrightarrow_{\mathrm{c}} (n_A - 1, n_{A_P} + 1, n_{A_{PP}})$ and $(n_A, n_{A_P}, n_{A_{PP}}) \leftrightarrow_{\mathrm{c}} (n_A - 1, n_{A_P}, n_{A_{PP}} + 1)$ for $n_A \geq 2$ and $n_A + n_{A_P} + n_{A_{PP}} = T_0$ because $\lambda_{A\to A_P}(n_A, n_{A_P}, n_{A_{PP}}) > 0, \lambda_{A_P\to A_{PP}}(n_A - 1, n_{A_P} + 1, n_{A_{PP}}) > 0$ and $\lambda_{A_{PP}\to A}(n_A - 1, n_{A_P}, n_{A_{PP}} + 1) > 0$, which imply that $(n_A, n_{A_P}, n_{A_{PP}}) \to_{\mathrm{a}} (n_A - 1, n_{A_P} + 1, n_{A_{PP}}) \to_{\mathrm{a}} (n_A - 1, n_{A_P}, n_{A_{PP}} + 1) \to_{\mathrm{a}} (n_A, n_{A_P}, n_{A_{PP}})$. As $\leftrightarrow_{\mathrm{c}}$ forms an equivalent class, any two states in $\{(x_1, x_2, x_3) \in \mathbb{Z}^3_{\geq 0} \mid x_1 \geq 1 \text{ and } x_1 + x_2 + x_3 = T_0\}$ communicate so it is irreducible when $T_0 \geq 2$. If $T_0 = 1$ then the set consists of a single element $(1, 0, 0)$, and it is irreducible itself.

Similarly, for the translated network in Fig. 1 we have that $(n_A, n_B) \leftrightarrow_{\mathrm{c}} (n_A + 1, n_B)$ and $(n_A, n_B) \leftrightarrow_{\mathrm{c}} (n_A, n_B + 1)$ for any $(n_A, n_B) \in \mathbb{Z}^2_{\geq 0}$ because $\lambda_{0\to A}(n_A, n_B) > 0$, $\lambda_{A\to A+B}(n_A + 1, n_B) > 0$ and $\lambda_{A+B\to 0}(n_A + 1, n_B + 1) > 0$, which imply that $(n_A, n_B) \to_{\mathrm{a}} (n_A + 1, n_B) \to_{\mathrm{a}} (n_A + 1, n_B + 1) \to_{\mathrm{a}} (n_A, n_B)$. As $\leftrightarrow_{\mathrm{c}}$ forms an equivalent class, any two states in $\mathbb{Z}^2_{\geq 0}$ communicate and hence $\mathbb{Z}^2_{\geq 0}$ is irreducible. The irreducibilities of the other two examples in Fig. 3a, g can be shown in similar ways.

Remark 5. By introducing a new factor $\omega(\mathbf{n})$, Theorem 2 generalizes Theorem 6.6 in [2], which is the most general version of the previous work [2]. In particular, if the function $\omega(\mathbf{n})$ for the factorization is $\theta(\mathbf{n})^{-1}$ then Theorem 2 becomes equivalent to Theorem 6.6 in [2]. This generalization is demonstrated by three biologically relevant examples in Fig. 3. For instance, $\omega(\mathbf{n}) = \theta(\mathbf{n})^{-1}\mathbf{1}_{\{\mathbf{n}\geq\mathbf{b}\}}$ in Fig. 3a with the nonzero vector $\mathbf{b} = (1, 0)$, so this factorization is not directly covered by the previously identified form, where $\omega(\mathbf{n}) = \theta(\mathbf{n})^{-1}$. However, the additional characteristic function is somehow negligible under a change of variable $\mathbf{n}' = \mathbf{n} - \mathbf{b}$ since the state space can be restricted on $\{\mathbf{n} \geq \mathbf{b}\}$. Nevertheless, the usefulness of our theorem is clearly demonstrated by the examples in Fig. 1 and Supplementary Figs. 3 and 4 .

SUPPLEMENTARY NOTE 4: CASTANET: COMPUTATIONAL PACKAGE FOR DERIVING STATIONARY DISTRIBUTION FORMULAE

Applying our theoretical framework (Fig. 1) has two practical difficulties. Translating a given network to a weakly reversible deficiency zero network (Fig. 1a) is not straightforward as prohibitively many candidates of translated networks often exist. Furthermore, unless propensity functions follow the generalized mass action kinetics, it is challenging to check whether the factorization condition holds (Fig. 1b) as it requires to solve associated recurrence relations. Thus, we have developed an open-source and publicly available MATLAB code (GitHub repository: http://github.com/Mathbiomed/CASTANET) that performs our theoretical analysis to derive stationary distributions. Specifically, the package checks two conditions: whether a given BRN can be made weakly reversible and of

zero deficiency after network translation, and whether the propensities of the translated network can be factorized as in Eq. (S2). If these two conditions are satisfied, the package then calculates the analytic formula for a stationary distribution. One can easily run our code by simply entering the source complexes, product complexes, and propensity functions of reactions.

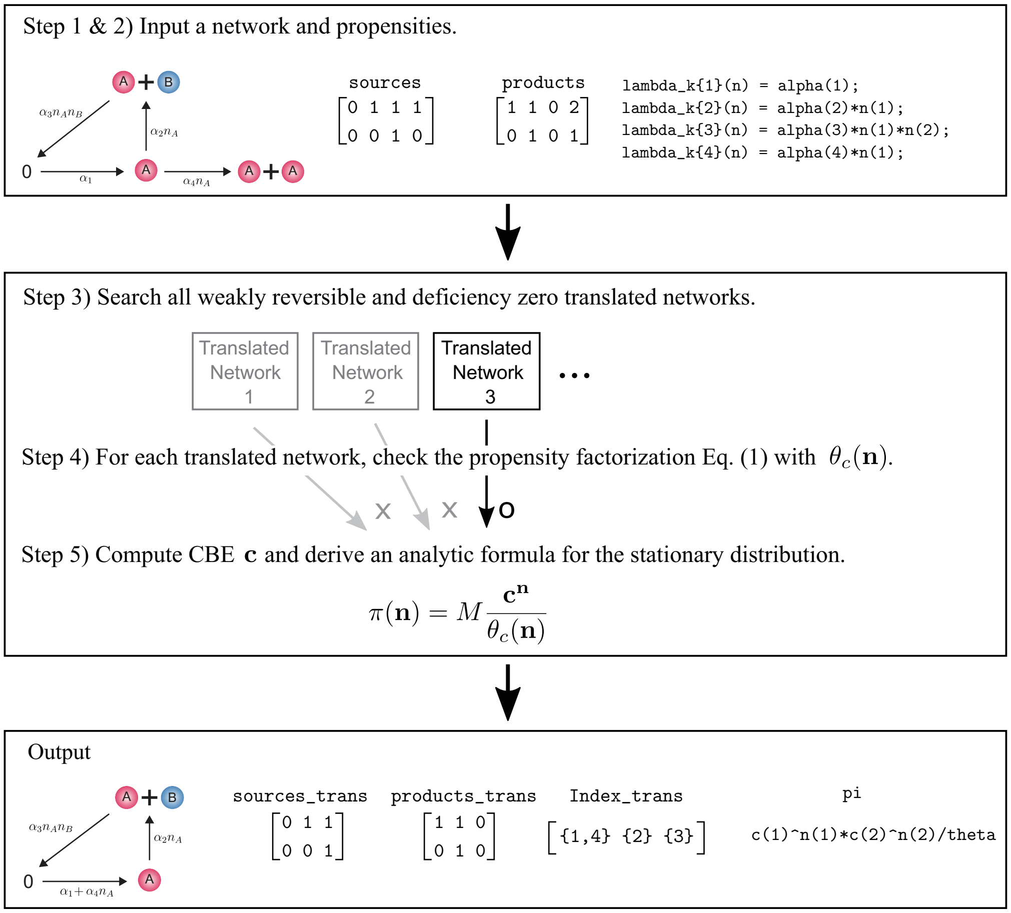

Supplementary Figure 2. A step-by-step schematic diagram of CASTANET. Here, $\theta_c(\mathbf{n})$ is a candidate for propensity factorization. See the manual for details.

Manual for the code

We explain how to enter the input and run the code `CRN_main.m` with our example in Fig. 1a as follows.

Step 1) Enter the source and the product complexes into `sources` and `products` as column vectors.

```
% Fig.1 example
sources = [0 0; 1 0; 1 1; 1 0]';
products = [1 0; 1 1; 0 0; 2 0]';
```

```
>> disp(sources)
     0 1 1 1
     0 0 1 0
```

```
>> disp(products)
```

```
1 1 0 2
0 1 0 0
```

The ith columns of `source` and `product` represent the source complex and the product complex of the ith reaction, respectively. For instance, the fourth columns of `source` ($[1,0]^{\mathrm{T}}$) and `product` ($[2,0]^{\mathrm{T}}$) describe the source and product complexes of the fourth reaction: $A(= 1A + 0B) \to A + A(= 2A + 0B)$.

Step 2) Enter the propensity functions of all reactions into the cell variable `lambda_k` and run the section `'Initialization of all input variables and parameters'` to set up all the input variables.

```
lambda_k{1}(n) = alpha(1);
lambda_k{2}(n) = alpha(2)*n(1);
lambda_k{3}(n) = alpha(3)*n(1)*n(2);
lambda_k{4}(n) = alpha(4)*n(1);
```

Note that the propensity functions do not need to follow the mass action kinetics. The symbolic variable `alpha(k)` represents the rate constants of the reactions. Users can also fix the values of `alpha(k)` (e.g., `alpha(1) = 3;`).

Step 3) Run the section `'Performing Network translation'` to obtain translated networks.

```
[Solution,Index] = CRN_translation(sources, products, 2);
```

The third input argument `2` means that the function searches all translated networks whose reaction order is at most two (i.e., bimolecular). It can be changed to any other positive integer. The ith row of `Solution` contains the source and product complexes of the ith translated network. Each element in the ith row of `Index` is a set of indices indicating which reactions in the original network are merged to form a reaction in the ith translated BRN. For instance, if the ij entry of `Index` is $\{1,3\}$, then this indicates that the first and third reactions in the original BRN were merged to form the jth reaction in the ith translated network. As the outcome, the number of identified weakly reversible deficiency zero translated networks is reported:

```
The number of weakly reversible deficiency zero translated networks is 2.
```

Step 4) Run the section `'Performing Propensity factorization'` to identify a translated network whose propensity functions satisfy the factorization condition (Eq. (S2)) among all the translated networks obtained in Step 3. Specifically, for each translated network, the code constructs a candidate ($\theta_{\mathrm{c}}(\mathbf{n})$) for the function $\theta(\mathbf{n})$ and checks the factorization conditions with the candidate $\theta_{\mathrm{c}}(\mathbf{n})$. This candidate is necessarily the desired function $\theta(\mathbf{n})$ if there exists a factorization (see the next subsection for details). The key function `CRN_theta_construction()` provides the candidate, and `CRN_check_factorization_condition()` examines whether the factorization condition (Eq. (S2)) holds. Since the example in Fig. 1 has a translated network satisfying the factorization condition, the code successfully finds the factorization and displays the following line:

```
The factorization condition holds for the translated network 1!
```

If none of the translated networks have the desired propensity factorization, the code displays the following line:

```
No translated network satisfying the factorization condition is
identified.
```

Step 5) If a translated network having the propensity factorization is found in Step 4, run the section `'Compute CBE and derive a stationary distribution pi(n)'` to compute a complex balanced equilibrium and analytically derive a stationary distribution. Then the code will provide the translated network and its stationary distribution formula, which is the same as the stationary distribution of the original network.

Output)

We have five outputs: `source_trans`, `product_trans`, `Index_trans`, `lambda_trans`, and `pi`. The first two outputs represent the stochiometric vectors of the source complexes and the product complexes in the translated network identified in Step 4. `Index_trans` is the row of `Index` corresponding to the translated network. `lambda_trans` contains the propensity functions of the translated network, and `pi` is a symbolic expression for the stationary distribution.

```
The source complexes of the translated network:
      0 1 1
      0 0 1

The product complexes of the translated network:
      1 1 0
      0 1 0

The index of the translated network:
      {1,4} {2} {3}
ni is the number of the ith species.
1st reaction propensity of the translated network:
alpha1 + alpha4*n1

2nd reaction propensity of the translated network:
alpha2*n1

3rd reaction propensity of the translated network:
alpha3*n1*n2

Analytic formula for the stationary distribution:
piecewise(~in((alpha1 - alpha4)/alpha4, 'integer') |
~(alpha1 - alpha4)/alpha4 in Dom::Interval([-n1], [-1]),
(alpha2^(n2 - n1)*alpha4^n1*gamma((alpha1 + alpha4*n1)/alpha4))/
(alpha3^n2*gamma(n1 + 1)*factorial(n2)*gamma(alpha1/alpha4)))
```

Here, `piecewise(condition, value)` means conditionally defined function in MATLAB. If `condition` holds then `value` is the function output. Since MATLAB symbolic expression uses `gamma(a+k+1)/gamma(a+1)` to represent $(a+1)(a+2)\cdots(a+k)$ and the domain of the gamma function does not contain the non-positive integers, the above complicated expression appears. The above expression certainly the same to the theoretical result.

Note that, technically, this is a formula for a stationary *measure*, not a distribution. To get the stationary distribution, the stationary measure needs to be normalized so that the sum of the probabilities over the state space is one, which is possible only when the formula is summable over the state space.

Underlying algorithm of the code

The code generates all possible network translations under a user-defined maximum reaction order (e.g., 1, 2, and 3 for unimolecular, bimolecular, and termolecular reaction networks, respectively). Among these translated networks, the code identifies weakly reversible deficiency zero networks. However, because there are sometimes prohibitively many translated networks (e.g., 864 candidates for the example in Fig. 1 with maximum reaction order 3), checking weak reversibility and deficiency for all translated network is extremely inefficient. In particular, it greatly increases computational cost to check weak reversibility and count the number of linkage classes via a connected components search algorithm (Tarjan's algorithm [12]). Thus, before performing this calculation, we first simply check whether a

translated network can have a desired property by using the following necessary conditions of being a weakly reversible deficiency zero network, which greatly reduce computational cost.

Theorem 3 (Necessary condition of a network to be weakly reversible after network translation)**.** *For a given biochemical reaction network, if there exists a weakly reversible translated network of the given network then for each species index i, one of the following two conditions holds.*

1. *The ith coordinates of the stoichiometric vectors of all reactions, $\nu'_{ik} - \nu_{ik}$, are zero. In other words, $\{\nu'_{i1} - \nu_{i1}, \nu'_{i2} - \nu_{i2}, \dots, \nu'_{ir} - \nu_{ir}\} = \{0\}$.*

2. *There exist both positive and negative numbers among the ith coordinates of the stoichiometric vectors of all reactions. In other words, the set $\{\nu'_{i1} - \nu_{i1}, \nu'_{i2} - \nu'_{i2}, \dots, \nu'_{ir} - \nu'_{ir}\}$ has both positive and negative elements.*

Proof. This can be proved by contradiction. Suppose that there exists a species index i such that the set of ith coordinates of all the stoichiometric vectors has only non-negative (non-positive) elements and has at least one positive (negative) element. Note that this is the negation of the above statement. Since the set of stoichiometric vectors is invariant under network translation, the weakly reversible translated network also has the same set of stoichoimetric vectors as the original network does. Note that every reaction in a weakly reversible network belongs to a closed cycle. However, the reaction whose stoichiometric vector has positive ith coordinate cannot be contained in any closed cycle because the ith coordinate of every other stoichiometric vector is non-negative. □

If the necessary condition is not satisfied for a given BRN, we do not need to check weak reversibility of all translated networks, which greatly reduces computational cost.

Theorem 4 (Necessary condition of a network to have zero deficiency)**.** *For a given biochemical reaction network, let n, l, and s be the number of complexes, the number of linkage classes, and the dimension of the subspace spanned by the stoichiometric vectors, respectively. If the deficiency of the network is zero, then $s + 1 \leq n \leq 2s$.*

Proof. The deficiency δ is given by $n - l - s$. Since the deficiency of the given network is zero, $n = l + s$. As long as the given network is not the empty network (i.e. $\mathcal{S} = \mathcal{C} = \mathcal{R} = \emptyset$), there exists at least one linkage class, so $1 \leq l$. Each linkage class consists of at least two complexes so $n(= l + s) \geq 2l$ and thus $l \leq s$. Therefore, $s + 1 \leq n = l + s \leq 2s$. □

This condition might appear useless because n can vary among translated network. Nevertheless, s is invariant under network translation, and furthermore, we no longer need to calculate the number of linkage classes l for the condition in Theorem 4, which requires to perform the graph search algorithm and thus takes high computational cost. Hence, by avoiding this calculation for translated networks that do not satisfy the necessary condition, we can greatly reduce computational cost.

After identifying a weakly reversible and of zero deficiency network, the code checks whether each translated network satisfies the factorization condition (Eq. (S2)) by constructing the explicit formula for a candidate $\theta_{\mathrm{c}}(\mathbf{n})$ for the function $\theta(\mathbf{n})$ in Eq. (S2). The candidate $\theta_{\mathrm{c}}(\mathbf{n})$ can be constructed as follows. We first derive the recurrence relation of the function $\theta(\mathbf{n})$. If there exists a desired propensity factorization, from the factorization conditions for reactions i and j at $\mathbf{n} - \nu_j$ and $\mathbf{n} - \nu_i$,

$$\lambda_i(\mathbf{n} - \nu_j) = \kappa_i \theta(\mathbf{n} - \nu_j)\omega(\mathbf{n} - \nu_j - \nu_i),$$
$$\lambda_j(\mathbf{n} - \nu_i) = \kappa_j \theta(\mathbf{n} - \nu_i)\omega(\mathbf{n} - \nu_i - \nu_j).$$

By dividing the above equations, we get the following recurrence relation:

$$\theta(\mathbf{n} - \nu_j) = \frac{\lambda_i(\mathbf{n} - \nu_j)/\kappa_i}{\lambda_j(\mathbf{n} - \nu_i)/\kappa_j} \times \theta(\mathbf{n} - \nu_i). \tag{S14}$$

Let $\mathbf{n}_0$ be a state satisfying $\lambda_k(\mathbf{n}_0) > 0$ for all k. Let us consider a sequence of pairs of source complexes, $(\nu_{a_1}, \nu_{b_1}), (\nu_{a_2}, \nu_{b_2}), \ldots, (\nu_{a_m}, \nu_{b_m})$, such that

$$\mathbf{n}_0 + \sum_{j=1}^{m} (\nu_{a_j} - \nu_{b_j}) = \mathbf{n}. \tag{S15}$$

This means that $\mathbf{n}_0 \to \mathbf{n}_0 + (\nu_{a_1} - \nu_{b_1}) \to \cdots \to \mathbf{n}_0 + \sum_{j=1}^{m}(\nu_{a_j} - \nu_{b_j}) = \mathbf{n}$ is a path from $\mathbf{n}_0$ to $\mathbf{n}$. For a pair of $\mathbf{n}$ and $\mathbf{n}_0$, in order to identify this type of paths, we use the row-style Hermite normal form, an analogue of reduced echelon form for a matrix over the integers. Specifically, non-zero rows of the Hermite normal form of the matrix whose jth row is $\nu_{j+1} - \nu_1$ form a basis for the lattice as $\nu_i - \nu_j = (\nu_i - \nu_1) - (\nu_j - \nu_1)$. Thus, by using this basis, one can identify a path from $\mathbf{n}_0$ to $\mathbf{n}$ [3, 5]. Note that such a path is not unique in general but it is enough to consider a single path (see below). We multiply Eq. (S14) along this path, and then we obtain

$$\theta(\mathbf{n}) = \prod_{j=1}^{m} \frac{\lambda_{a_j}(\mathbf{n}_0 + \sum_{i=1}^{j}(\nu_{a_i} - \nu_{b_i}))/\kappa_{a_j}}{\lambda_{b_j}(\mathbf{n}_0 + \sum_{i=1}^{j-1}(\nu_{a_i} - \nu_{b_i}))/\kappa_{b_j}} \times \theta(\mathbf{n}_0). \tag{S16}$$

Note that the RHS of Eq. (S16) can be obtained by substituting $\mathbf{n}$ in Eq. (S14) with $\mathbf{n} + \nu_i$ and one can assume $\theta(\mathbf{n}_0) = 1$ without loss of generality as the factorization holds up to constants.

For a special case, CASTANET uses a more efficient way to identify $\theta(\mathbf{n})$. Specifically, if the propensity functions of a translated network follow the generalized stochastic mass action kinetics and all the complexes consist of zero or one species, then CASTANET constructs the function $\theta(\mathbf{n})$ by using Eq. (S7).

Based on this expression for $\theta(\mathbf{n})$, the code constructs the candidate $\theta_{\mathrm{c}}(\mathbf{n})$ using Eq. (S16) with a path from $\mathbf{n}_0$ to $\mathbf{n}$. If there exists a desired function $\theta(\mathbf{n})$ for propensity factorization then it must be the same as the candidate $\theta_{\mathrm{c}}(\mathbf{n})$ because both should be expressed as Eq. (S16). Thus, the propensity factorization condition (Eq. (S2)) can be checked using $\theta_{\mathrm{c}}(\mathbf{n})$. In the package, `CRN_find_elementary_path()` generates a path from $\mathbf{n}_0$ to $\mathbf{n}$, `CRN_theta_construction()` constructs $\theta_{\mathrm{c}}(\mathbf{n})$, and `CRN_check_factorization_condition()` examines the factorization condition. The functions `CRN_find_elementary_path()`, `CRN_find_elementary_function()` and `CRN_solve_sym_linear()` are auxiliary functions to preprocess the input variables into appropriate forms for the construction code. See `README.md` in the GitHub repository for details. Finally, a complex balanced equilibrium of the deterministic mass action model for the translated network is determined by solving the algebraic equation for the complex balanced equilibrium with the function `CRN_compute_cbe()`.

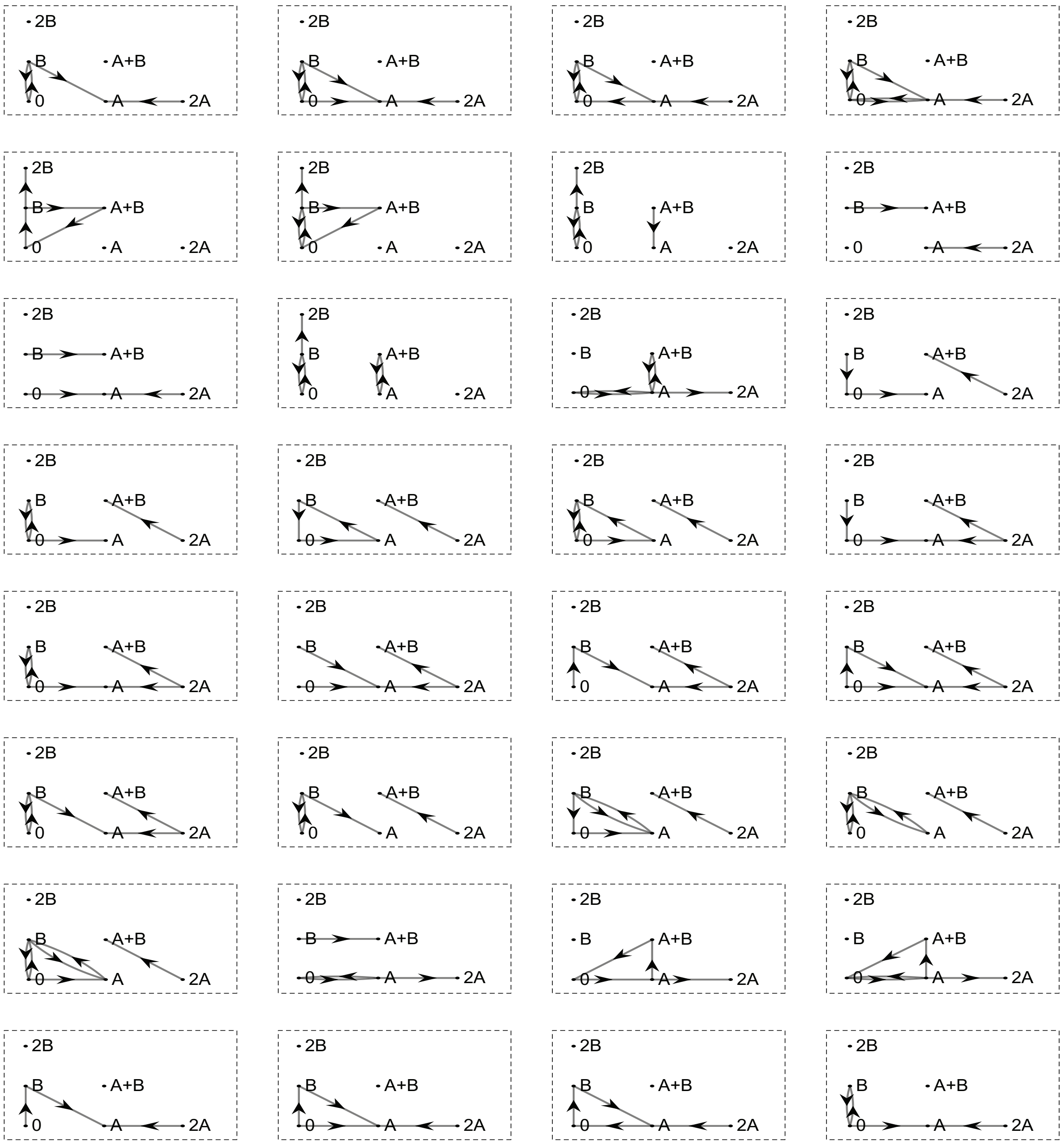

Supplementary Figure 3. Our computational package CASTANET identifies BRNs with two species whose stationary distribution can be analytically derived. Specifically, CASTANET translates these networks to be weakly reversible and of zero deficiency and then finds factorizations for the propensity functions of the translated networks as in Eq. (S8), thus calculating their stationary distributions. The figure shows the first 32 networks identified by our package among randomly selected networks with two species (A and B) and at most bimolecular reactions (e.g., $A + B \to A$). More examples can be found in https://github.com/Mathbiomed/CASTANET. All kinetics are assumed to follow the mass action kinetics. All rate constants are set to be one to reduce complexity while arbitrary rate constants are allowed to derive stationary distributions.

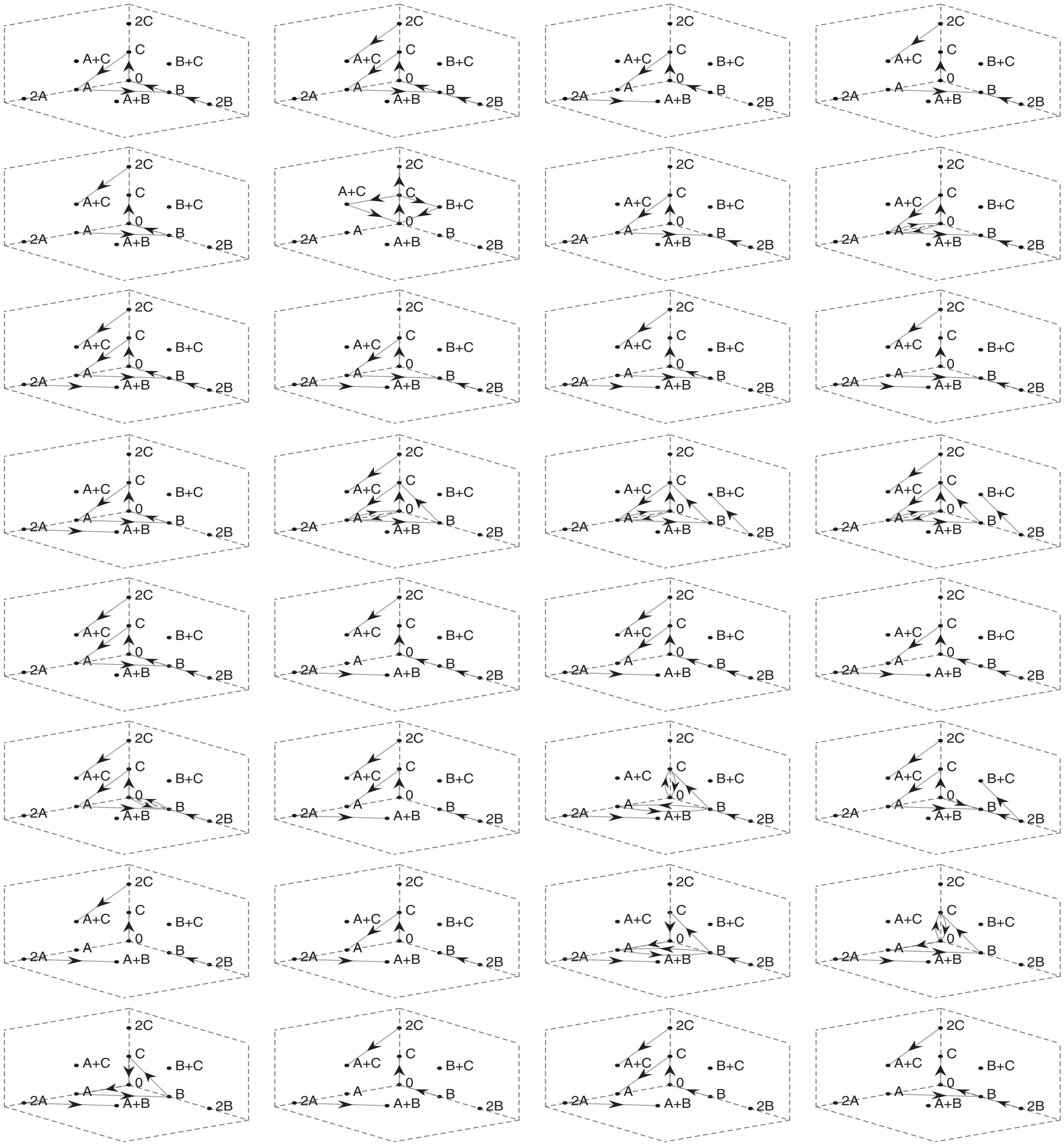

Supplementary Figure 4. Our computational package CASTANET identifies BRNs with three species whose stationary distribution can be derived. Specifically, CASTANET translates these networks to be weakly reversible and of zero deficiency and then finds factorizations for the propensity functions of the translated networks as in Eq. (S8), thus calculating their stationary distributions. The figure shows the first 32 networks identified by our package among randomly selected networks with three species (A, B, and C) and at most bimolecular reactions (e.g., $A + B \to C$). More examples can be found in https://github.com/Mathbiomed/CASTANET. All kinetics are assumed to follow the mass action kinetics. All rate constants are set to be one to reduce complexity while arbitrary rate constants are allowed to derive stationary distributions.

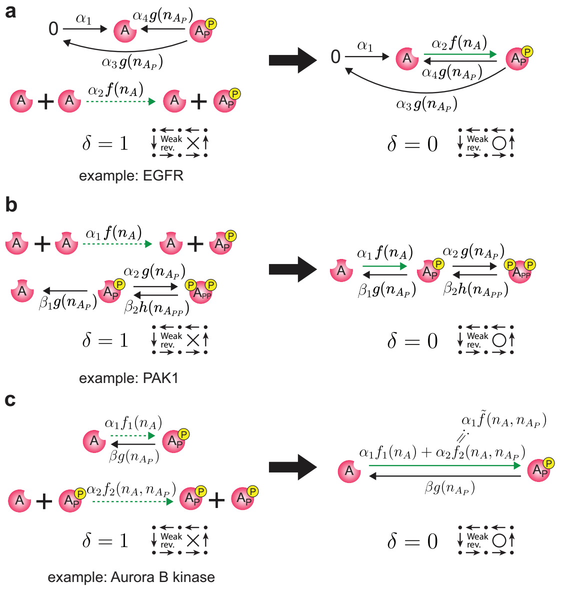

d

$$\pi(n_A, n_{A_P}) = M \frac{\left(\frac{\alpha_1}{\alpha_2} + \frac{\alpha_1\alpha_4}{\alpha_2\alpha_3}\right)^{n_A} \left(\frac{\alpha_1}{\alpha_3}\right)^{n_{A_P}}}{\prod_{j=b_A+1}^{n_A} f(j) \prod_{j=b_{A_P}+1}^{n_{A_P}} g(j)} \quad \text{if } n_A \geq b_A \text{ and } n_{A_P} \geq b_{A_P}$$

e

$$\pi(n_A, n_{A_P}, n_{A_{PP}}) = M \frac{(\beta_1\beta_2)^{n_A} (\alpha_1\beta_2)^{n_{A_P}} (\alpha_1\alpha_2)^{n_{A_{PP}}}}{\prod_{j=b_A+1}^{n_A} f(j) \prod_{j=b_{A_P}+1}^{n_{A_P}} g(j) \prod_{j=b_{A_{PP}}+1}^{n_{A_{PP}}} h(j)}$$

$$\text{if } n_A \geq b_A,\ n_{A_P} \geq b_{A_P}, n_{A_{PP}} \geq b_{A_{PP}}, \text{ and } n_A + n_{A_P} + n_{A_{PP}} = T_0$$

f

$$\pi(n_A, n_{A_P}) = M \frac{\beta^{n_A} \alpha_1^{n_{A_P}}}{\prod_{j=b_A+1}^{n_A} \tilde{f}(j, n_A + n_{A_P} - j) \prod_{j=b_{A_P}+1}^{n_{A_P}} g(j)}$$

$$\text{if } n_A \geq b_A, n_{A_P} \geq b_{A_P}, \text{ and } n_A + n_{A_P} = T_0$$

Supplementary Figure 5. Stationary distributions of autophosphorylation networks with non-elementary reactions (e.g., Hill functions). **a, b, c** Autophosphorylation networks with non-elementary propensity functions. The propensity functions f, f_1, f_2, g, and h can be Michaelis-Menten or Hill functions, which are often used to describe autophosphorylation, phosphatase-mediated dephosphorylation, and proteasomal degradation. The propensity functions f, f_1, f_2, g, and h can be Michaelis-Menten or Hill functions, which are often used to describe autophosphorylation, phosphatase-mediated dephosphorylation, and proteasomal degradation. In fact, $f(n_A)$ can be other functions as long as there exists b_A such that $f(n_A) > 0$ if and only if $n_A > b_A$. For the Michaelis-Menten and Hill-type functions, $b_A = 0$ because $f(n_A) > 0$ if and only if $n_A > b_A = 0$. The restriction $f(n_A) > 0$ if and only if $n_A > b_A$ means simply that the propensity is positive when there is a large enough number of A molecules (i.e., $n_A > b_A$), and the value of b_X is, in turn, the minimum number of species A in the recurrent state. This indicates that our method can be applied to any typical propensity function f for these networks. Similarly, $\tilde{f}, g$, and h can be other functions satisfying the natural positivity condition (e.g, $g(n_{A_P}) > 0$ if and only if $n_{A_P} > b_{A_P}$). **d, e, f** Stationary distributions of the autophosphorylation networks with the non-elementary reactions. Note that the stationary distributions of the networks in Fig. 3 are special cases of these formulae.

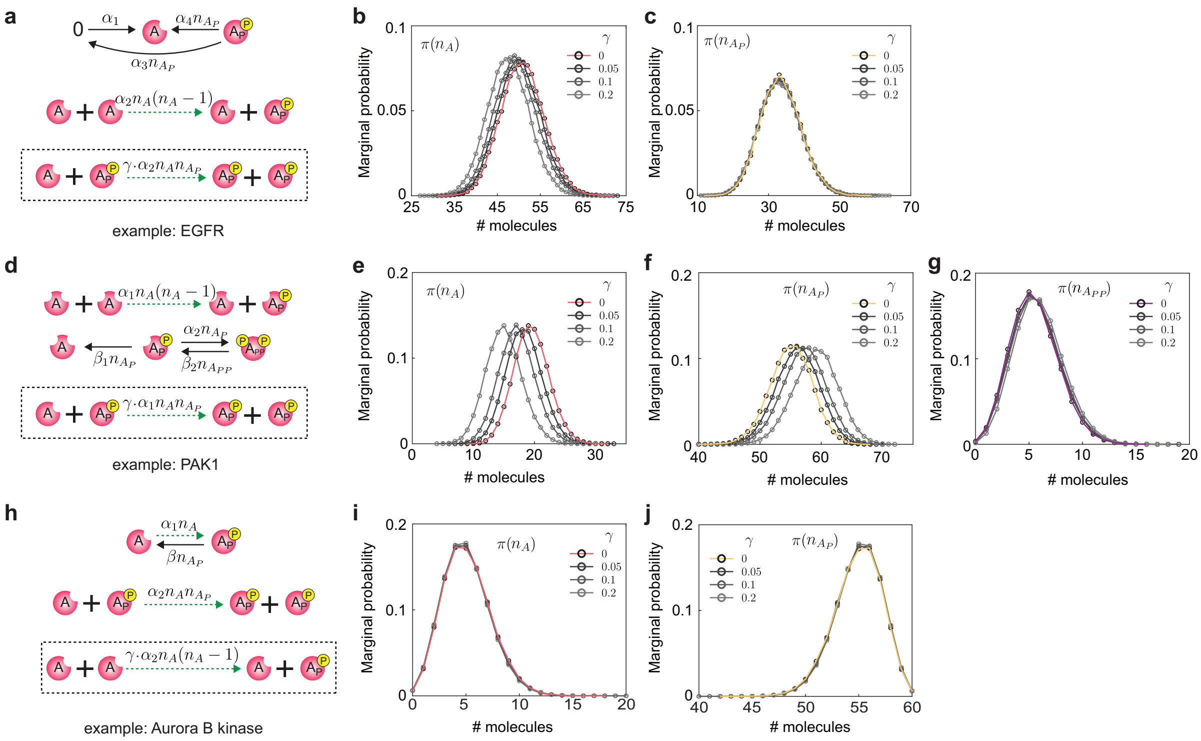

Supplementary Figure 6. The stationary distributions of modified autophosphorylation networks with additional trans-autophosphorylation reactions (dotted boxes). These can be approximated by the stationary distributions of the original networks as long as the relative magnitude (γ) of the rate constant of a new trans-autophosphorylation reaction (dotted box) is sufficiently small compared to that of the original trans-autophosphorylation reaction. **a** Trans-autophosphorylation reaction $A + A_P \to A_P + A_P$ is added to the original network (Fig. 3a). Due to this modification, although the modified network can be translated to a weakly reversible deficiency zero network, a desired propensity factorization does not exist. **b** Nevertheless, the stationary distributions $\pi(n_A)$ of the modified network ($\gamma = 0.05, 0.1, 0.2$, gray lines) calculated from stochastic simulations can be closely approximated by the stationary distribution of the original network ($\gamma = 0$, colored line), which is derived by our method (Fig. 3b, c). **c** Even the stationary distributions $\pi(n_{A_P})$ of the modified network are nearly identical to those of the original network. **d** Similar to **a**, adding $A + A_P \to A_P + A_P$ makes their propensities unable to be factorized as in Eq. (S2) even after network translation. **e, f, g** The stationary distributions, $\pi(n_A)$, $\pi(n_{A_P})$, and $\pi(n_{A_{PP}})$ of the modified network can be approximated by the stationary distributions of the original network ($\gamma = 0$), which are obtained by our method (Fig. 3e, f). **h** Another trans-autophosphorylation reaction $A + A \to A + A_P$ is added to the original network (Fig. 3g). **i,j** The stationary distributions $\pi(n_A)$ and $\pi(n_{A_P})$ of the modified network are nearly identical to the stationary distributions of the original network ($\gamma = 0$). Note that the stationary distribution of the modified network in **h** can still be analytically derived because there exists a desired propensity factorization for this modified network after network translation. For each example, 10^5 simulations were performed using the Gillespie algorithm. See Fig. 3 for the values of parameters.

SUPPLEMENTARY NOTE 5: STATIONARY DISTRIBUTIONS OF THE GENETIC TOGGLE SWITCH WITH SLOW PROMOTER KINETICS (FIG. 4)

To obtain the probabilities of the gene G^A is active (ρ_{act}) and repressed (ρ_{rep}), we can construct the reduced-order Markov chain infinitesimal generator Λ_r:

$$[\Lambda_r]_{ij} = \left\{ \begin{array}{ll} \varepsilon^{-1}\mathbb{E}\left[\lambda_{ij}|\text{gene state } j\right] & \text{for } i \neq j \\ -\varepsilon^{-1}\sum_{k\neq j}\lambda_{kj} & \text{for } i = j \end{array} \right\},$$

where λ_{ij} is the propensity of the reaction that changes the gene state from j to i, and ε is the ratio between the fast and slow timescale. The gene state $1, 2, 3$, and 4 represent (G^A_{act}, G^B_{act}), (G^A_{act}, G^B_{rep}), (G^A_{rep}, G^B_{act}), and (G^A_{rep}, G^B_{rep}), respectively, where G^A_{act} represents $n_{G^A_{act}} = 1$ and $n_{G^A_{rep}} = 0$, and G^A_{rep} represents $n_{G^A_{act}} = 0$ and $n_{G^A_{rep}} = 1$, and it is defined in the same manner for the gene G^B. Then λ_{21} is the propensity of the reaction $A_P + G^B_{act} \to G^B_{rep}$, which changes the gene state from (G^A_{act}, G^B_{act}) to (G^A_{act}, G^B_{rep}). Thus, $[\Lambda_r]_{21} = \varepsilon^{-1}\mathbb{E}\left[\lambda_{21}|(G^A_{act}, G^B_{act})\right] = l_b\mathbb{E}\left[n_{A_P}|(G^A_{act}, G^B_{act})\right]$ because $\lambda_{21} = \varepsilon \cdot l_b n_{A_P} n_{G^B_{act}}$, and $n_{G^B_{act}} = 1$. Since the conditional moment of n_{A_P} is solely determined by the state of the gene G^A, $[\Lambda_r]_{21} = l_b\mathbb{E}\left[n_{A_P}|G^A_{act}\right]$. Repeating the similar computations, we get the infinitesimal generator

$$\Lambda_r = \begin{bmatrix} -l_b\mathbb{E}\left[n_{A_P}|G^A_{act}\right] - k_b\mathbb{E}\left[n_{B_P}|G^B_{act}\right] & l_u & k_u & 0 \\ l_b\mathbb{E}\left[n_{A_P}|G^A_{act}\right] & -l_u - k_b\mathbb{E}\left[n_{B_P}|G^B_{rep}\right] & 0 & k_u \\ k_b\mathbb{E}\left[n_{B_P}|G^B_{act}\right] & 0 & -k_u - l_b\mathbb{E}\left[n_{A_P}|G^A_{rep}\right] & l_u \\ 0 & k_b\mathbb{E}\left[n_{B_P}|G^B_{rep}\right] & l_b\mathbb{E}\left[n_{A_P}|G^A_{rep}\right] & -k_u - l_u \end{bmatrix}.$$

Note that Λ_r is Metzler i.e., all the entries are non-negative except for those on the diagonal, and $\mathbf{1}^T\Lambda_r = 0$. Hence, the Perron-Frobenius theorem [6] implies that there exists a positive eigenvector $\rho = (\rho_{(act,act)}, \rho_{(act,rep)}, \rho_{(rep,act)}, \rho_{(rep,rep)})$ corresponding to the eigenvalue 0. Furthermore, we can uniquely determine the eigenvector ρ using the normalization: $\rho_{(act,act)} + \rho_{(act,rep)} + \rho_{(rep,act)} + \rho_{(rep,rep)} = 1$. Here, $\rho_{(act(,rep),act(,rep))}$ is the probability that the gene state is $(G^A_{act(,rep)}, G^B_{act(,rep)})$ [1]. From this eigenvector, we can derive the approximation of the full (i.e., unconditional) mariginal stationary distribution of fast variable A_P :

$$\begin{aligned} \pi(n_{A_P}) \approx & \rho_{(act,act)}\pi(n_{A_P}|G^A_{act}, G^B_{act}) + \rho_{(act,rep)}\pi(n_{A_P}|G^A_{act}, G^B_{rep}) \\ & + \rho_{(rep,act)}\pi(n_{A_P}|G^A_{rep}, G^B_{act}) + \rho_{(rep,rep)}\pi(n_{A_P}|G^A_{rep}, G^B_{rep}), \end{aligned} \tag{S17}$$

where the approximation becomes more accurate as the timescale separation is larger and becomes exact when $\varepsilon \to 0$. Since the conditional distribution of the A_P solely depends on the state of the gene G^A, it can be reduced as follows:

$$\pi(n_{A_P}) \approx (\rho_{(act,act)} + \rho_{(act,rep)})\pi(n_{A_P}|G^A_{act}) + (\rho_{(rep,act)} + \rho_{(rep,rep)})\pi(n_{A_P}|G^A_{rep}).$$

Finally, the probabilities that the gene G^A is active (ρ_{act}) and repressed (ρ_{rep}) are given by

$$\rho_{act} = \rho_{(act,act)} + \rho_{(act,rep)} \quad \text{and} \quad \rho_{rep} = \rho_{(rep,act)} + \rho_{(rep,rep)}.$$

The full marginal stationary distributions of all other fast variables: A, B, B_P, can also be obtained by computing the linear combination of the conditional stationary distributions of each fast variable, similar to Eq. (S17) (Supplementary Fig. 7). More details on the derivation of the reduced-order Markov chain are discussed in [1].

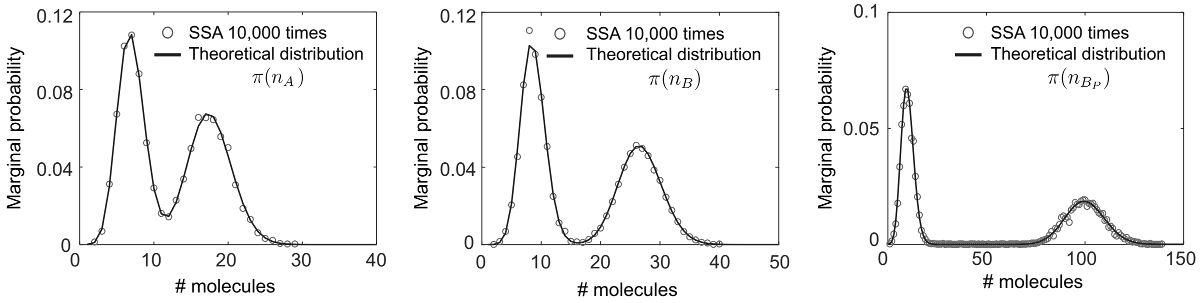

Supplementary Figure 7. Full marginal stationary distributions formulae (solid lines) and the simulation results obtained with 10^4 times Gillespie algorithms (dots) for A, B, B_P in Fig. 4. See Fig. 4 for the values of parameters.

SUPPLEMENTARY REFERENCES

[1] M. Ali Al-Radhawi, Domitilla Del Vecchio, and Eduardo D. Sontag. Multi-modality in gene regulatory networks with slow promoter kinetics. *PLoS Computational Biology*, 15(2):1–27, 2019.

[2] David F. Anderson, Gheorghe Craciun, and Thomas G. Kurtz. Product-form stationary distributions for deficiency zero chemical reaction networks. *Bulletin of Mathematical Biology*, 72(8):1947–1970, 2010.

[3] Henri Cohen. A course in computational algebraic number theory. *Graduate texts in Math.*, 138:88, 1993.

[4] Martin Feinberg. Complex balancing in general kinetic systems. *Archive for Rational Mechanics and Analysis*, 49(3):187–194, 1972.

[5] Ankit Gupta and Mustafa Khammash. Determining the long-term behavior of cell populations: A new procedure for detecting ergodicity in large stochastic reaction networks. *IFAC Proceedings Volumes*, 47(3):1711–1716, 2014. 19th IFAC World Congress.

[6] D. Hinrichsen and N. K. Son. μ-analysis and robust stability of positive linear systems. *Applied Mathematics and Computer Science*, 8:253–268, 1998.

[7] Fritz Horn. Necessary and sufficient conditions for complex balancing in chemical kinetics. *Archive for Rational Mechanics and Analysis*, 49(3):172–186, 1972.

[8] Fritz Horn and Roy Jackson. General mass action kinetics. *Archive for Rational Mechanics and Analysis*, 47(2):81–116, 1972.

[9] F. Kelly. *Reversibility and Stochastic Networks*. Wiley, New York, 1979.

[10] J.R. Norris. *Markov Chains*. Cambridge Series in Statistical and Probabilistic Mathematics. Cambridge University Press, 1998.

[11] Eduardo D Sontag. Structure and stability of certain chemical networks and applications to the kinetic proofreading model of t-cell receptor signal transduction. *IEEE transactions on automatic control*, 46(7):1028–1047, 2001.

[12] Robert Tarjan. Depth-first search and linear graph algorithms. *SIAM journal on computing*, 1(2):146–160, 1972.
