# Supplementary material for: Derivation of stationary distributions of biochemical reaction networks via structure transformation
Source: Commun Biol. 2021 May 24;4:620. doi: 10.1038/s42003-021-02117-x (PMC8144570; doi:10.1038/s42003-021-02117-x)
Supplement: Supplementary file 3 — Description of Additional Supplementary Files [file 42003_2021_2117_MOESM3_ESM.pdf]

Description of Additional Supplementary Files

File Name: Supplementary Data 1

Description: Simulation data underlying plots shown in Figures 3 and 4.
