# Supplementary material for: Derivation of stationary distributions of biochemical reaction networks via structure transformation
Source: Commun Biol. 2021 May 24;4:620. doi: 10.1038/s42003-021-02117-x (PMC8144570; doi:10.1038/s42003-021-02117-x)
Supplement: Supplementary file 1 — Peer Review File [file 42003_2021_2117_MOESM1_ESM.pdf]

This manuscript has been previously reviewed at another Nature Research journal. This document only contains reviewer comments and rebuttal letters for versions considered at Communications Biology.
